# Temporary carriage of bovine coronavirus and bovine respiratory syncytial virus by fomites and human nasal mucosa after exposure to infected calves

**DOI:** 10.1186/s12917-018-1335-1

**Published:** 2018-01-22

**Authors:** Veslemøy Sunniva Oma, Thea Klem, Madeleine Tråvén, Stefan Alenius, Britt Gjerset, Mette Myrmel, Maria Stokstad

**Affiliations:** 1 Department of Production Animal Clinical Sciences, Norwegian University of Life Sciences, P.O. Box 8146 Dep, 0033 Oslo, Norway; 2 Department of Clinical Sciences, Swedish University of Agricultural Sciences, 75007 Uppsala, Sweden; 3 Norwegian Veterinary Institute, P.O. Box 750 Sentrum, 0106 Oslo, Norway; 4 Department of Food Safety and Infection Biology, Norwegian University of Life Sciences, P.O. Box 8146 Dep, 0033 Oslo, Norway

**Keywords:** Indirect transmission, Virus infectivity, Biosecurity, Bovine respiratory disease, Human nasal mucosa, Cattle

## Abstract

**Background:**

In order to prevent spread of the endemic pathogens bovine coronavirus (BCoV) and bovine respiratory syncytial virus (BRSV) between herds, knowledge of indirect transmission by personnel and fomites is fundamental. The aims of the study were to determine the duration of viral RNA carriage and the infectivity of viral particles on fomites and human nasal mucosa after exposure to BCoV and BRSV. During two animal infection experiments, swabs were collected from personnel (nasal mucosa) and their clothes, boots and equipment after contact with calves shedding either virus. Viral RNA was quantified by RT-qPCR or droplet digital RT-PCR (RT-ddPCR), and selected samples with high levels of viral RNA were tested by cell culture for infectivity.

**Results:**

For BCoV, 46% (*n* = 80) of the swabs from human nasal mucosa collected 30 min after exposure were positive by RT-qPCR. After two, four and six hours, 15%, 5% and 0% of the swabs were positive, respectively. Infective virions were not detected in mucosal swabs (*n* = 2). A high viral RNA load was detected on 97% (*n* = 44) of the fomites 24 h after exposure, and infective virions were detected in two of three swabs. For BRSV, 35% (*n* = 26) of the human nasal mucosa swabs collected 30 min after exposure, were positive by RT-ddPCR, but none were positive for infective virions. Of the fomites, 89% (*n* = 38) were positive for BRSV RNA 24 h after exposure, but all were negative for infective viruses.

**Conclusions:**

The results indicate that human nasal mucosa can carry both BCoV and BRSV RNA after exposure to virus shedding calves, but the carriage seems short-lived and the transmission potential is likely limited. High viral loads on contaminates fomites 24 h after exposure to infected animals, and detection of infective BCoV, indicate that contaminated fomites represent a significant risk for indirect transmission between herds.

## Background

Bovine coronavirus (BCoV) and bovine respiratory syncytial virus (BRSV) are contagious pathogens detrimentally affecting production and animal welfare in the cattle industry. The viruses are part of the bovine respiratory disease complex and are endemic worldwide. BRSV and BCoV can cause epidemics of respiratory disease and additionally BCoV cause diarrhea in calves and adult cattle (winter dysentery) [1–4]. The traditional way of handling and preventing these diseases is through metaphylactic antibiotic treatment, use of vaccines, or changes in management to improve calf health in herds [5]. The within-herd prevalence and morbidity of BCoV and BRSV infections are high [6, 7] and once the virus enters a herd, circulation is difficult to mitigate. An additional preventive strategy is therefore to reduce inter-herd transmission of virus. Movement of live animals between herds is an important transmission route [8]. If this risk is under control, the next question concerns the contribution of indirect spread of virus between herds. Indirect spread can occur via e.g. personnel travelling between herds, their clothes or equipment.

Important risk factors for indirect spread are the level of virus contamination of relevant surfaces and the infectivity of the viruses. Enveloped respiratory viruses like BCoV and BRSV are generally fragile outside the host [9]. However, as related viruses like human respiratory syncytial virus (HRSV) and human coronavirus 229E remain infective for several hours on contaminated surfaces like countertops and surgical gloves [10, 11], there is a potential for indirect transmission. Epidemiological studies also point out the importance of indirect transmission; Ohlson et al. found that lack of boot provision for visitors was a risk factor for infections with both viruses [12] and Toftaker et al. found that a herd’s BCoV and BRSV antibody status was influenced by the status of its neighboring herds [8].

Human nasal mucosa might also be a vector for inter-herd virus transmission, as traffic of personnel between herds is common. Carriage of BCoV and BRSV in human nostrils has not been studied. Generally, there are few studies on indirect transmission of these viruses, and no experimental studies have been performed. Molecular methods and virus isolation in cell culture can be used to study the level of virus carriage and infectivity, which are determinants for virus transmission. Combined, these methods provide sensitive quantification of viral genomes and assessment of virus infectivity.

Consequently, the aim of the present study was to investigate whether personnel (nostrils) and fomites carry viral RNA and infective viruses after exposure to BCoV or BRSV infected animals.

## Methods

### Study design and animal experiments

The present study was performed during two animal experiments, one with BCoV and one with BRSV, and during a field outbreak of winter dysentery. Swabs were rubbed in the nostrils of personnel and on their coats, boots, wristwatches and stethoscopes at different time points after animal contact, and examined for viral RNA and infective viruses.

The BCoV experiment was conducted in 2014 at the Swedish University of Agricultural Sciences (SLU) as described by Oma et al. [13]. A total of ten bull calves between six and twelve weeks of age were included, six were Swedish red and white, three were Swedish Holstein and one Swedish mountain breed. Briefly, four calves at SLU were exposed to a group of six calves brought in from SLU’s research farm that experienced an outbreak of winter dysentery. The field outbreak was confirmed by RT-qPCR and serology to have been caused by BCoV. After comingling for 24 h, the calves were housed in the isolation unit within their original groups of four and six animals. As six of the calves were naturally exposed to BCoV in the field, the dates of infection were unknown. The presented contamination study was conducted within a three week period while the calves showed signs of disease and shed virus as detected by RT-qPCR. The number of BCoV RNA copies in nasal swabs from the ten calves varied between log_10_ 2.9 and 10.4 (mean of log_10_ 6.9) during the study period.

The BRSV experiment took place at the Norwegian Veterinary Institute in 2015 (to be published). A total of eight Norwegian Red calves between two and four months of age were included, six bulls and two heifers. Briefly, six of the calves were infected after contact with two calves inoculated with a field isolate of BRSV, O4-4B/N-11 [14]. The calves were housed in isolation units in groups of four including one inoculated calf. The contamination study was conducted on three different days within one week while the calves showed signs of respiratory disease and shed virus. The number of BRSV RNA copies in nasal swabs from the calves varied between log_10_ 2.7 and 8.1 (mean of log_10_ 5.6) during the study period.

Both experiments were conducted in line with the ARRIVE guidelines for planning and reporting in vivo experiments and the concept of the 3R’s (Reduction, Replacement and Refinement) [15, 16]. In both experiments, efforts were made to minimize the stress and discomfort for the animals. The animals were closely monitored and medical treatment were administered in line with national Norwegian and Swedish recommendations for treatment of pneumonia and diarrhea in calves.

### Exposure procedure and sampling schemes

Table 1 presents an overview of exposed personnel and fomites. During ten minutes, the personnel handled and examined animals that showed clinical signs and shed either BCoV or BRSV. In the BCoV experiment, swabs were collected from human nostrils prior to and 0.5, 2, 4 and 6 h after exposure to the animals. The BRSV experiment included only a single time point (0.5 h), as viral RNA was not detected in nasal swabs collected during a BRSV pilot study.

**Table 1:** Overview of personnel and fomites that were sampled after exposure to virus shedding animals

	BCoV^a^ experiment	BRSV^b^ experiment	Winter dysentery outbreak in dairy herd
No. of animals	10	8	300
No. of persons	16	12	19
Personnel			
No. of challenges	86	26	19
Hours between exposure and sampling	−0.5, 0.5, 2, 4 and 6	0.5	0.5, 2, 4
Fomites			
No. of challenges	44	38	–
No. and types	12 rubber coats, 16 rubber boots, 8 stethoscopes, 8 wrist watches	19 rubber coats, 19 rubber boots	–
Hours between exposure and sampling	0, 2 and 24	2 and 24	–

Sample collection was performed during two animal experiments and one outbreak of winter dysentery (caused by BCoV). ^a^BCoV – bovine coronavirus, ^b^BRSV - bovine respiratory syncytial virus

Clean boots, coats, wristwatches and stethoscopes were used. After exposure to the animals, boots were rinsed in lukewarm water until visually clean and left to dry. All fomites were stored at 16–18 °C, in a room separate from the animals.

### Sampling procedure

A detailed protocol was developed for collection of material from fomites and human nostrils. The same person collected all the material from fomites in each experiment, and instructed the personnel that took part in the human mucosa trial. Specimens were collected with ESwab™ (Copan, Brescia, Italy) and stored in 1 ml of Liquid Amies medium. Gloves were used throughout the experiments.

Specimens from human nasal mucosa were collected by rotating a swab inside one nostril for a couple of seconds. When a person was sampled more than once, the left and right nostrils were sampled alternately. Sampling of fomites was performed by moistening the tip of the swab with Amies medium before lightly rubbing a defined area (5 cm × 10 cm of coats and boots) without visible contamination. For wristwatches and stethoscopes, the area was approximately 3 cm × 3 cm and 2 cm × 5 cm, respectively. At later time points, new areas were sampled. After sample collection, swabs were stored at 4 °C for no more than two hours and thereafter at −70 °C until use.

### RNA extraction and quantification of viral genomes

#### BCoV

RNA was extracted from 140 μl of the Amies medium by the QIAamp Viral RNA Mini QIAcube kit (Qiagen, Hilden, Germany) according to the manufacturer’s instructions, eluted in 50 μl buffer and stored at −80 °C. RT-qPCR was performed, in duplicates for nasal swabs, using RNA UltraSense™ One-Step Quantitative RT-PCR System (Invitrogen, MA, USA) and the target was an 85 bp fragment of the M protein gene [17]. Two μl of RNA was used in a total volume of 20 ul containing 200 nM each of forward and reverse primer and 250 nM TaqMan probe. The thermal profile included an RT step at 55 °C for 30 min followed by 95 °C for 2 min and thereafter 40 cycles of 15 s at 95 °C and 1 min at 60 °C. RT-qPCR was run in a Stratagene Mx3005p™ (Agilent Technologies, CA, USA) and each run included a positive (RNA from the nostril of a BCoV positive trial calf) and negative control (water).

Positive swabs from human nasal mucosa were subjected to Kaplan-Meier survival analysis in Stata (Stata SE/14, Stata Corp., College Station, TX, USA). The function shows the cumulative survival, i.e. carriage of BCoV RNA over time, which descends as personnel turns BCoV RNA negative. As the exact time-point a person turned negative was unknown, the mid-point between the last positive and the first negative sample was used in the analysis [18].

In order to estimate the number of BCoV RNA genome copies (GC), a standard curve was prepared using tenfold dilutions of a plasmid containing the BCoV target sequence. The BCoV RNA positive control was aliquoted and included in every RT-qPCR plate as a calibrator to adjust for inter-plate variation. The number of GC in clinical samples was calculated using the formula from Livak and Schmittgen [19]:

$$ {Q}_s={Q_c}^{\ast }{\left(1+E\right)}^{-\left({Ct}_s-{Ct}_c\right)} $$

Where *Q*_*s*_ *= sample RNA copy number, Q*_*c*_ *= calibrator RNA copy number, Ct*_*s*_ *= sample Ct value, Ct*_*c*_ *= calibrator Ct value* and *E = efficiency of target amplification.*

The standard curve covered the range from 10.8 to 1.08 × 10^10^ copies. The curve showed a strong linear relationship with a high coefficient of determination (R^2^ = 0.996) and a high amplification efficiency (E = 0.965). The limit of quantification represented log_10_ 3.6 BCoV GC per swab from human nasal mucosa and fomites.

#### BRSV

RNA was extracted from 200 μl of Amies medium, using the automated NucliSens easyMAG protocol (Biomérieux, Marcy l’Etoile, France), according to the manufacturer’s instructions. Quantification of BRSV genomes was conducted in duplicate, with Bio-Rad’s QX200 ddPCR System (droplet digital PCR). Each run included a positive (RNA from the nostril of a BRSV positive trial calf) and negative control (water). Droplet generation and transfer of droplets were as described by the manufacturer. The One-Step RT-ddPCR Advanced Kit for Probes (BioRad, CA, USA) and 2 μl RNA were used. The sequence of primers and probe (5’FAM and BHQ1 as quencher) was as described [20], targeting a 123 bp region of the BRSV N gene. Primers and probe concentrations were as recommended by the kit manufacturer and with the following cycling conditions; 50 °C for 60 min, 95 °C for 10 min and 40 cycles of 95 °C for 30 s and 60 °C for 1 min. The ramp rate was set to 2 °C/s. Data processing and absolute quantification of BRSV genomes per μl RNA was performed with QuantaSoft Version 1.7 (BioRad).

Half-life calculation for BRSV RNA carriage was unattainable due to single sampling.

### Testing of virus infectivity

#### BCoV

Virus infectivity was tested in five samples with the highest level of BCoV RNA, using integrated cell culture RT-qPCR; swabs from a wristwatch, a stethoscope and a coat collected 24 h after exposure, and from two human nostrils, collected 30 min after exposure. The swab medium was diluted 1:10 in Dulbecco’s Modified Eagle Medium (DMEM, Thermo Fisher Scientific, DE, USA) and added, in duplicate wells, to monolayers of 4-days-old human rectal tumor cells (HRT-18, ATCC CRL-11663) in a 24-well plate. Positive control (cultivated BCoV from a calf in the experiment), positive control in Amies medium and negative controls (cells only) were included. After incubation at 37 °C for one hour, the inoculum was removed, the cells washed and DMEM with 1% fetal calf serum (FCS) and antibiotics (5000 IU penicillin and 5 mg streptomycin/ml) was added. Simultaneously, cells were harvested from one parallel well of each sample as a time zero replication control. After three days incubation at 37 °C in 5% CO_2_, cells were harvested from the remaining wells and RNA extracted with Qiazol (Qiagen), chloroform phase separation (mixed 1:1 with 70% ethanol), and RNeasy Mini Kit column (Qiagen). The amount of RNA was measured using Nanodrop (Thermo Fisher Scientific) and equal amounts analyzed by BCoV RT-qPCR run in duplicates as described. Relative quantification of target RNA from incubated and time zero replication control cells was performed using the standard curve.

#### BRSV

BRSV infectivity was tested in ten swabs showing the highest level of viral RNA by RT-ddPCR; eight swabs from coats and two from human nostrils, collected 24 h and 30 min after exposure, respectively. Fetal bovine turbinate cells (courtesy of Swedish Veterinary Institute) propagated in Eagle’s minimal essential medium (BioWhittaker, Belgium) in 96 well plates were incubated with 50 μl filtered swab samples for 30 min. Medium with 2% FCS was added, the plates were incubated at 37 °C in 5% CO_2_ and the supernatant passaged after seven days. Samples were cultivated in duplicates with positive (cultivated BRSV from a calf in the experiment) and negative controls (cells only). The cells were observed for cytopathic effect (CPE) and infection visualized by direct immunofluorescence test using FITC Moab a-BRSV (Bio-X Diagnostics, Rochefort, Belgium). Culture supernatants were harvested and tested by the BRSV RT-ddPCR as described.

## Results

### Viral RNA in human nasal mucosa

**Fig. 1:**
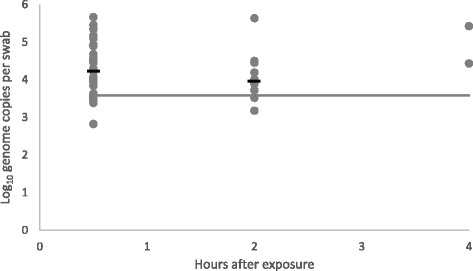
Bovine coronavirus (BCoV) contaminated human nasal mucosa. Log_10_ genome copies of BCoV per positive swab. Personnel had close contact for at least ten minutes with calves shedding BCoV. Swabs were taken from human nostrils at different time points after exposure to the calves. The grey line shows the limit of quantification and the black short lines indicate median genome copies per positive swab

**Fig. 2:**
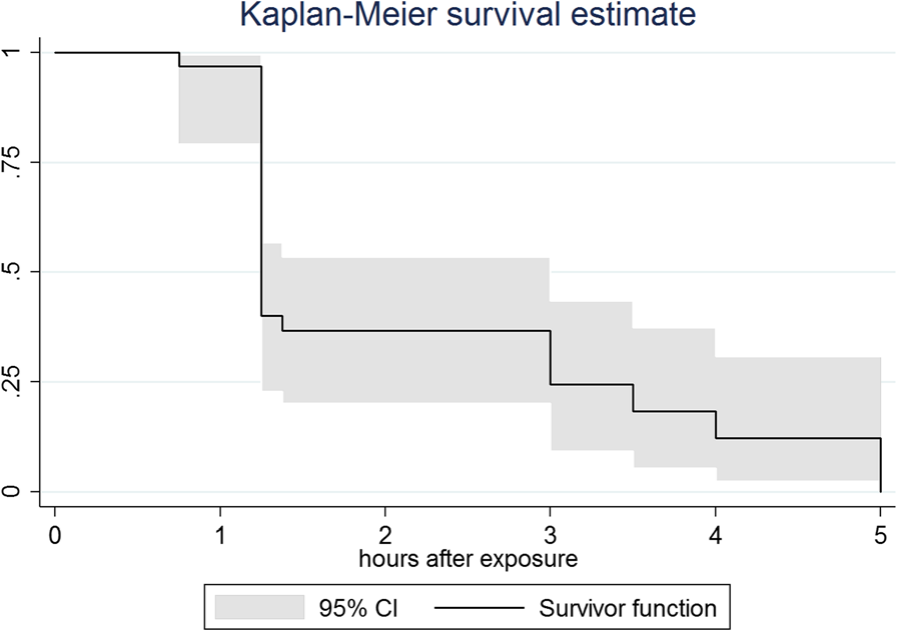
Kaplan-Meier survival function for BCoV RNA carriage in human nasal mucosa

The positive controls were consistently positive throughout the analyses, and all negative controls were negative. No viral RNA was detected in human nasal mucosa that was sampled prior to exposure to animals, however, positives were found among samples collected after exposure (Table 2). The number of BCoV GC per swab is shown in Fig. 1. Estimated half-life of BCoV RNA carriage was less than 90 min and the estimated longest persistence was five hours (Fig. 2). The positive BRSV swabs contained between log_10_ 1.3 to 3.3 genome copies.

**Table 2:** BCoV and BRSV RNA in human nasal swabs

	BCoV^a^ experiment		BRSV^b^ experiment		Winter dysentery outbreak	
Hours between exposure and sampling	Total no. of swabs	No. of positive swabs (%)	Total no. of swabs	No. of positive swabs (%)	Total no. of swabs	No. of positive swabs (%)
−0.5	67	0	ND^c^	ND	ND	ND
0.5	80	37 (46%)	26	9 (35%)	7	1 (14%)
2	68	10 (15%)	ND	ND	1	0
4	38	2 (5%)	ND	ND	12	0
6	28	0	ND	ND	ND	ND
24	11	0	ND	ND	ND	ND
Total	292	49 (17%)	26	9 (35%)	20	1 (5%)

RT-qPCR and droplet digital RT-PCR results in swabs from the nasal cavity of personnel before and after exposure to BCoV or BRSV infected calves

^a^BCoV – bovine coronavirus

^b^BRSV – bovine respiratory syncytial virus

^c^ND = Not done

### Viral RNA on fomites

**Fig. 3:**
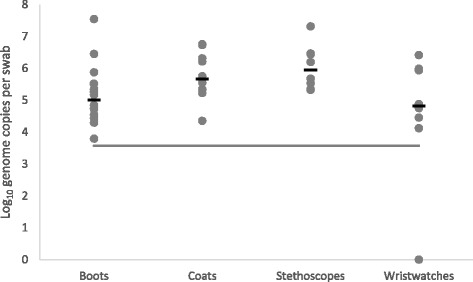
Bovine coronavirus (BCoV) contaminated fomites. Log_10_ numbers of genome copies per swab taken 24 h after exposure to BCoV-infected calves. The grey line shows the limit of quantification and the black short lines indicate median genome copies per swab

**Fig. 4:**
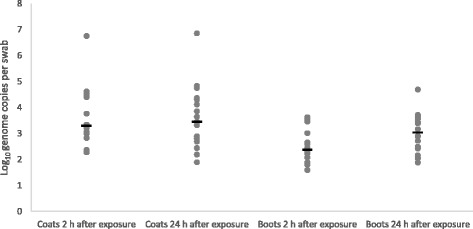
Bovine respiratory syncytial virus (BRSV) contaminated fomites. Log_10_ numbers of genome copies in positive swabs taken after exposure to BRSV-infected calves. The black short lines indicate median genome copies per swab

The positive controls were consistently positive throughout the analyses, and all negative controls were negative. BCoV RNA was detected on all boots, coats and stethoscopes, and on seven out of eight wristwatches, 24 h after exposure. The eighth watch was positive for BCoV RNA 15 min and two hours after exposure. The copy numbers of BCoV RNA 24 h after exposure are presented in Fig. 3. BRSV RNA was detected on 18 out of 19 boots sampled after two hours and 16 out of 19 boots after 24 h. For the coats, 17 out of 19 were positive two hours after exposure, and 18 out of 19 were positive after 24 h. There were minor differences in BRSV RNA copy numbers between samples collected 2 and 24 h after exposure and no tendency of reduction in copy numbers (Fig. 4).

### Virus infectivity

#### BCoV

RT-qPCR results from cells inoculated with swab material from a wristwatch or a stethoscope indicated a 1000-fold increase in the number of RNA-copies after three days of incubation. Cells inoculated with swab material from human nostrils and from a rubber coat showed no increase in viral RNA during incubation. Positive virus controls were positive, and the Amies medium showed no inhibition of virus replication. No BCoV RNA was detected in negative control wells.

#### BRSV

No CPE was seen in cells incubated with swab material or with passaged material and RT-ddPCR results did not indicate any virus replication after two passages in the cells. Positive control wells were positive, and negative control wells were negative.

## Discussion

This is the first time BCoV and BRSV contamination of personnel and fomites has been described. The PCR results indicate that fomites (like clothes, boots, wristwatches and stethoscopes) exposed to virus pose an infection risk to cattle. For BCoV in particular, fomites seem to represent a high risk, as virus isolation detected infective viruses after 24 h. Consequently, measures to prevent inter-herd transmission should include actions against indirect spread of virus.

As high copy numbers of viral RNA on fomites indicated a transmission potential, further investigations were performed in order to assess whether the detected RNA could represent infective viruses. Although infectivity ideally should be studied in live animals, cell culture was used due to practical, ethical and economic reasons. Virus isolation in cell culture may have a low sensitivity [21], but the use of integrated cell culture RT-qPCR increases the possibility of detecting infective viruses [22]. Using this method, we showed that visually clean surfaces of fomites can carry infective BCoV for at least 24 h after exposure to infected animals.

As reviewed by La Rosa et al., related coronaviruses and HRSV can be transmitted by fomites in addition to direct transmission through droplets and aerosols [23]. It is therefore plausible that BCoV and BRSV could be transmitted between farms via personnel and fomites. Even if protective clothing is used and changed between herds, personnel might constitute a risk of virus transmission as human nasal mucosa could be a potential hideaway for infective viruses. In addition, BCoV has been isolated from a diarrheic child and is most likely the ancestor of a related human pathogen [24–26], thus the ability to replicate in cells in the human nasal mucosa cannot be excluded. Human nasal mucosa was therefore studied in place of skin, oral mucosa or hair that could also act as passive vectors for the viruses.

Sellers et al. have shown that human nasal mucosa is a possible vehicle for foot-and-mouth-disease virus even when a high level of biosecurity is implemented [27]. This was refuted by Amass et al. who found a low risk of virus transmission by personnel after hand wash and change of outerwear [28]. Wright et al. found a low risk of prolonged human nasal carriage of the virus [29]. In the present study, we aimed to study whether human nasal mucosa is a possible vehicle for transmission of BCoV and BRSV. Based on our results, this is a possibility, but the low level of viral RNA and the failure to detect infective virus after a few hours, indicate a low risk of virus transmission from human nasal mucosa.

In the present study, personnel was sampled during an experimental setting and during an outbreak of winter dysentery in the field. The results showed that nasal carriage of BCoV in humans was less common in the outbreak situation than during the animal experiment. Factors that could have influenced the amount of virus in the two settings were differences in virus exposure, degree of contact between animals and personnel and environmental conditions. Other factors could be repeated swabbing of the same nostril, nose touching and nose blowing.

The finding that neither BCoV nor BRSV could be cultivated from human nasal swabs resembles the rapid inactivation on skin for respiratory syncytial virus [11] and human coronavirus 229E [30]. This could be due to substances or microorganisms in the mucosa that neutralize or inactivate the virus. Although there is a chance of underestimating the risk, due to e.g. freezing and thawing, dilution and filtering of the samples, the virus transmission potential of mucosa is probably low. There were no sign of BCoV replicating in human nasal mucosa, as the amount of BCoV RNA found were low and declining over time.

Despite the general view that enveloped viruses are fragile outside the host, several coronaviruses remain infective after drying on surfaces for more than 24 h as reviewed by Otter et al. [31]. The present study indicates that BCoV has a similar property. Infective BRSV, on the other hand, was not detected in any of the samples, which was similar to HRSV after drying on surfaces for seven hours [11]. Studies of HRSV survival in cell culture medium and aerosols also showed a higher inactivation rate compared to coronaviruses [32–35]. This suggests that BRSV is more susceptible than BCoV to degradation by environmental factors, and that the importance of indirect BRSV transmission after 24 h, is probably low. As demonstrated by Mullis et al. [36], viral infectivity is more rapidly lost than viral RNA.

For both viruses, the viral RNA level recovered from boots was lower than from coats, possibly due to the rinsing with water. However, as high genome copy numbers remained, rinsing might not be sufficient to prevent virus transmission. This is supported by epidemiologic data that show an increased risk of seropositivity for BRSV and BCoV in herds that do not provide boots to visitors [37]. The present BCoV experiment indicated that also stethoscopes and wristwatches could serve as vehicles. These items are often brought between farms without cleaning/disinfection, and can carry infective virus particles for at least 24 h after exposure to infected cattle.

## Conclusions

Personnel pose a risk in inter-herd transmission of BRSV and BCoV when bringing fomites between herds. In order to control the spread of these viruses, biosecurity measures should be implemented, including herd-specific clothing and equipment and washing/disinfection of fomites. Although personnel may carry the viruses intra-nasally for shorter periods of time, the relative importance of contaminated mucosa for indirect transmission is less than that of contaminated fomites.

## Abbreviations

BCoV: Bovine coronavirus
BRSV: Bovine respiratory syncytial virus
CPE: Cytopathic effect
Ct: Cycle threshold
DMEM: Dulbecco’s Modified Eagle Medium
FCS: Fetal calf serum
GC: Genome copies
HRSV: Human respiratory syncytial virus
HRT: Human rectal tumor cells
RT-ddPCR: Reverse transcription droplet digital polymerase chain reaction
RT-qPCR: Quantitative reverse transcriptase polymerase chain reaction
SLU: Swedish University of Agricultural Sciences
μl: Microliter
